# Supplementary material for: Combined BSA-Seq Based Mapping and RNA-Seq Profiling Reveal Candidate Genes Associated with Plant Architecture in Brassica napus
Source: Int J Mol Sci. 2022 Feb 23;23(5):2472. doi: 10.3390/ijms23052472 (PMC8910715; doi:10.3390/ijms23052472)
Supplement: Supplementary file 1 [file ijms-23-02472-s001.zip › Supplementary Figures.pdf]

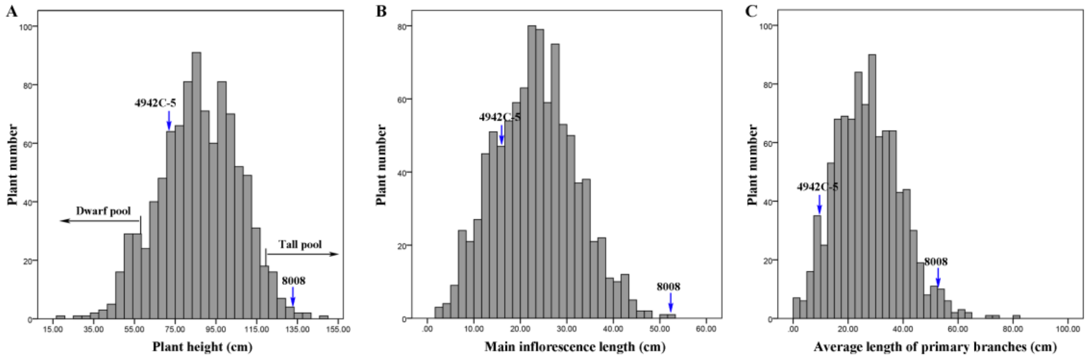

Figure S1. (A) Frequency distribution of plant height in the BC_8 population. (B) Frequency distribution of main inflorescence length in the BC_8 population. (C) Frequency distribution of average length of primary branches in the BC_8 population.

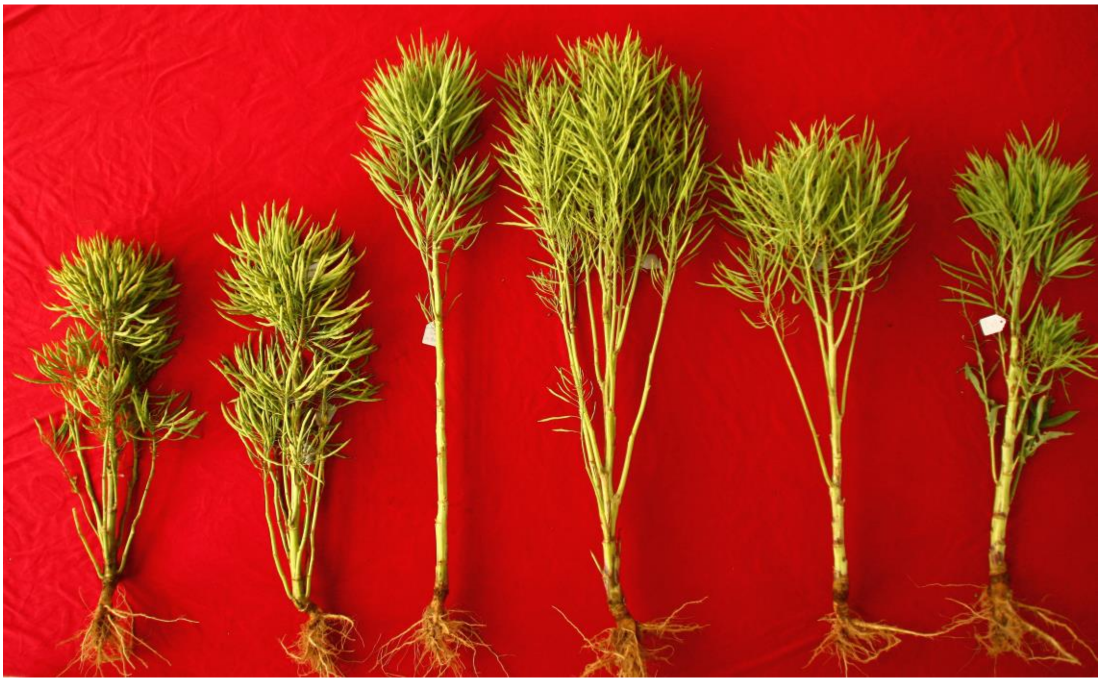

Figure S2. Plant architecture of offspring derived from crosses between 4942C-5 and 8008.

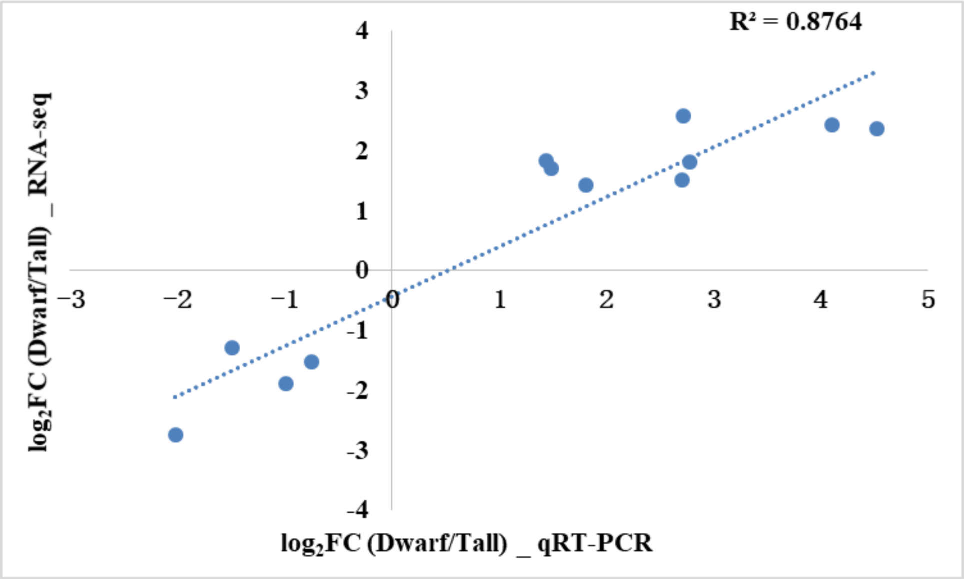

Figure S3. Correlation of the gene expression ratios between qRT-PCR and RNA-seq data. Expression abundance of 12 selected DEGs is presented as the ratio of Tall and Dwarf plants. Data represent mean values ± SE of three biological replicates and three technical replicates of each sample.

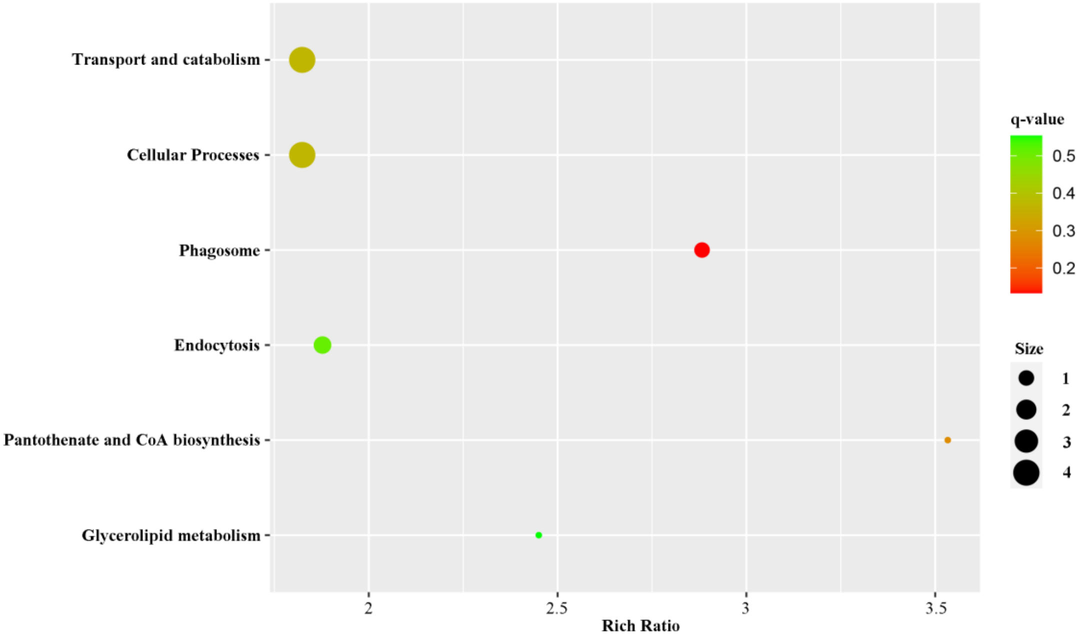

Figure S4. KEGG pathway enrichment of 542 DEGs in the candidate intervals.
